# *Ob/ob* Mouse Livers Show Decreased Oxidative Phosphorylation Efficiencies and Anaerobic Capacities after Cold Ischemia

**DOI:** 10.1371/journal.pone.0100609

**Published:** 2014-06-23

**Authors:** Michael J. J. Chu, Anthony J. R. Hickey, Sherry Tagaloa, Linda Zhang, Anna J. Dare, Julia R. MacDonald, Mee-Ling Yeong, Adam S. J. R. Bartlett, Anthony R. J. Phillips

**Affiliations:** 1 Department of Surgery, University of Auckland, Auckland, New Zealand; 2 School of Biological Sciences, University of Auckland, Auckland, New Zealand; 3 Maurice Wilkins Centre for Biodiscovery, University of Auckland, Auckland, New Zealand; 4 Department of Anatomical Pathology, Auckland City Hospital, Auckland, New Zealand; 5 New Zealand Liver Transplant Unit, Auckland City Hospital, Auckland, New Zealand; University of Colorado Denver, United States of America

## Abstract

**Background:**

Hepatic steatosis is a major risk factor for graft failure in liver transplantation. Hepatic steatosis shows a greater negative influence on graft function following prolonged cold ischaemia. As the impact of steatosis on hepatocyte metabolism during extended cold ischaemia is not well-described, we compared markers of metabolic capacity and mitochondrial function in steatotic and lean livers following clinically relevant durations of cold preservation.

**Methods:**

Livers from 10-week old leptin-deficient obese (*ob/ob*, n = 9) and lean C57 mice (n = 9) were preserved in ice-cold University of Wisconsin solution. Liver mitochondrial function was then assessed using high resolution respirometry after 1.5, 3, 5, 8, 12, 16 and 24 hours of storage. Metabolic marker enzymes for anaerobiosis and mitochondrial mass were also measured in conjunction with non-bicarbonate tissue pH buffering capacity.

**Results:**

*Ob/ob* and lean mice livers showed severe (>60%) macrovesicular and mild (<30%) microvesicular steatosis on Oil Red O staining, respectively. *Ob/ob* livers had lower baseline enzymatic complex I activity but similar adenosine triphosphate (ATP) levels compared to lean livers. During cold storage, the respiratory control ratio and complex I-fueled phosphorylation deteriorated approximately twice as fast in *ob/ob* livers compared to lean livers. *Ob/ob* livers also demonstrated decreased ATP production capacities at all time-points analyzed compared to lean livers. *Ob/ob* liver baseline lactate dehydrogenase activities and intrinsic non-bicarbonate buffering capacities were depressed by 60% and 40%, respectively compared to lean livers.

**Conclusions:**

Steatotic livers have impaired baseline aerobic and anaerobic capacities compared to lean livers, and mitochondrial function indices decrease particularly from after 5 hours of cold preservation. These data provide a mechanistic basis for the clinical recommendation of shorter cold storage durations in steatotic donor livers.

## Introduction

Hepatic steatosis is the most common chronic liver disease in the Western world and is frequently associated with the metabolic syndrome [1]. With the increasing prevalence of metabolic syndrome, the incidence of hepatic steatosis is expected to rise, as is the frequency of encountering steatotic donor livers for transplantation [1]. Hepatic steatosis in donor livers appears to negatively impact graft function [2]. Moderate (30–60%) and severe (>60%) steatosis is associated with higher rates of graft failure [2], [3].

One of the proposed mechanisms for explaining the poor outcome of steatotic livers post-transplantation is the presence of an underlying metabolic dysfunction, which impacts mitochondrial function (MF) and leads to impaired energy production [4]. Mitochondria supply the vast majority of cellular energy as adenosine triphosphate (ATP) via oxidative phosphorylation (OXPHOS) [5]. Intact MF is therefore critical for cellular homeostasis as they provide the majority of ATP with a small contribution from anaerobic substrate metabolism. Conversely, mitochondrial dysfunction may lead to impaired cellular homeostasis and cell death [6]. In the transplantation setting, the sum of any underlying functional metabolic impairment or acquired mitochondrial injuries resulting from steatosis may lead to the inability of hepatocytes to rapidly regain full function post-transplantation.

Cold ischaemic time is known to be a significant determinant of graft outcome and prolonged cold ischemia is associated with decreased graft survival [2]. Mitochondrial dysfunction following reperfusion has been shown to occur with prolonged cold ischemia [4] and steatotic livers [7]. This is likely to impair ATP synthesis during the critical time of its high demand during recovery after reperfusion. Although experimental studies suggest that mitochondrial dysfunction and decreased ATP levels occur after prolonged (>18 hours) cold ischaemia [4], [7], a thorough investigation of steatotic liver mitochondrial function exposed to a more clinically relevant 9–18 hours of cold ischemic storage time, is lacking. No studies have also tested the impact of hepatic steatosis on hepatic anaerobic function, or on pH buffering capacity during cold storage. Furthermore, there has been no prior parallel assessment of hepatic MF and ATP production rate during cold ischemia. The current understanding of mitochondrial dysfunction has been mostly based on absolute ATP level measurements, so there is still a significant deficit in the understanding of the underlying mitochondrial impairment in hepatic steatosis subjected to cold ischaemia [8]. Additional studies of steatotic liver MF after cold ischemia in physiological experimental conditions are required to improve our understanding of this disease process in order to inform management practices of the steatotic liver.

Here we use the *ob/ob* mouse steatotic liver model, and this study is the first detailed time-course study of steatotic liver MF, ATP production, anaerobic and pH buffering capacity during clinically relevant durations of cold storage ischemia.

## Materials and Methods

### Ethics statement

All experiments were conducted in accordance with the regulations provided by the Guide for Care and Use of Laboratory Animals, and were approved by the University of Auckland Animal Ethics Committee.

### Animals

All reagents were purchased from Sigma-Aldrich (New South Wales, Australia) unless otherwise specified. Male Lep^(−/−)^ (*ob/ob*) and lean (control) C57/BL6J mice were kept under a 12∶12-hour light/dark cycle (50–70% humidity, 20–24°C) with *ad libitum* access to standard chow (Teklad 2018, Harlan, Madison, WI) and water for 10 weeks (n = 9 mice/group).

### Blood glucose measurement, glucose tolerance test (GTT) and insulin tolerance test (ITT)

Blood glucose was measured on blood from a tail stab with a 23 g needle. Mice were fasted overnight for 12–13 hours at age 8 and 9 weeks for GTT and ITT, respectively. After intra-peritoneal injection of glucose (2 mg/g) or insulin (0.25 mU/g), GTT and ITT were performed on with collection of tail blood glucose at 15, 30, 60, and 120 minutes after injection, respectively. Mice were re-fed after GTT and ITT.

### Tissue collection

Ten-week old animals were anesthetized by intra-peritoneal injection of phenobarbitone. Tissue collections were performed 5–7 days after ITT to minimize any acute effect of ITT on liver metabolic status. The time of tissue collection was similar for all mice (∼0900–1000). Multiple 4 mm^3^ liver biopsies were collected and immediately stored in 1 ml of ice-cold University of Wisconsin solution until MF assessment (UW; in mM: 100 Potassium lactobionate, 25 KH_2_PO_4_, 5 MgSO_4_, 30 Raffinose, 5 Adenosine, 3 Glutathione, 1 Allopurinol, 50 g/L Hydroxyethyl starch). The remaining liver was bisected then fixed in 10% neutral-buffered formalin or snap-frozen in liquid nitrogen and stored at −80°C for later analysis.

### Histology

Baseline (Time 0) formalin-fixed and paraffin-embedded liver samples were sectioned at 4 µm for Haematoxylin and Eosin (H&E) staining while baseline frozen liver samples were sectioned at 10 µm for Oil-Red O staining. An experienced liver histopathologist (MY) blinded to the treatment group examined the sections using a commonly used clinical grading system [3].

### Permeabilized tissue preparation

The liver was removed from UW solution and placed into ice-cold mitochondrial respiration media consisting of (in mM: 0.5 EGTA, 3 MgCl_2_, 60 K-lactobionate, 20 taurine, 10 KH_2_PO_4_, 110 sucrose and 1 mg/mL bovine serum albumin in 20 HEPES (pH 7.0 at 37°C), and mechanically permeabilized into 0.1–0.2 mm^3^ pieces with two pairs of forceps as described previously [9].

### Respiration assays and time-course analysis

Mitochondrial respiration was measured after liver removal at 1.5 (“baseline”), 3, 5, 8, 12, 16 and 24 hours, on matched independently stored tissue segments. True (t = 0) baseline analyses were not able performed due to the inherent time required for sample transport, preparation and permeabilization, so a first time-point (1.5 hours) was chosen as a baseline time to provide the same starting time-point for all sample analysis. Respiration was measured in 2-mL chambers using an OROBOROS Oxygraph 2K (Anton Paar, Graz, Austria), at 37°C in mitochondrial respiration media, with a calculated saturated oxygen concentration of 190 nmol O_2_ per mL at 100 kPa barometric pressure. The permeabilised liver samples blotted dry on lint-free paper weighed 2–3 mg, and the weight-specific oxygen flux [pmol O_2_ (s.mg wet wt)^−1^] was calculated using the DatLab 4 analysis software.

A multiple substrate-inhibitor titration protocol was used to explore relative contributions of complex I (CI), complex II (CII) and CI+CII in the electron transport system (ETS) [10]. Respiration states were defined according to Gnaiger [11], where LEAK respiration (*L*) and OXPHOS was the flux measured before and after addition of adenosine diphosphate (ADP), respectively. The assay was commenced with the addition of CI substrates: glutamate (10 mM) and malate (5 mM) [*L*_CI_] followed by ADP (1.25 mM) [OXPHOS-CI]. Cytochrome C (10 µM) and pyruvate (10 mM) were then added. Succinate (10 mM) was added, thereby activating CI and CII simultaneously and driving parallel electron input into the ETS (OXPHOS-CI,CII). Atractyloside (0.25 mM) was added to inhibit adenine nucleotide translocase and thereby to assess CI+CII leak (*L*_Atra_). Three incremental additions of carbonylcyanide *p*-trifluoromethoxy-phenylhydrazone (final concentration 1.5 µM) were made to uncouple respiration as a measure of ETS capacity. The CI inhibitor rotenone (1 µM) was added to isolate flux to CII. Malonate (15 mM) and antimycin A (5 µM) were then added, and the artificial electron donor systems of N,N,N0,N0-tetramethyl-p-phenylendiamine (0.5 mM) with ascorbate (2 mM) were added to assay complex IV. Integrity of tissue preparations and comparison of coupling efficiencies were assessed from the respiratory control ratio (RCR, OXPHOS-CI/*L*_CI_ ratio).

### ADP and ATP analysis

Basal hepatic ADP and ATP levels were measured in homogenates using NADH-coupled assays and followed at 340 nm [12]. Frozen liver samples (50–100 mg) were homogenised in 0.3 ml of 3 M perchloric acid followed by addition of 1.25 ml of 1 mM EDTA and mixed for 5–10 minutes at 4°C before centrifugation at 20,000 g for 1 minute (4°C). One ml of supernatant was then mixed with 330 µl of 2 M KHCO_3_ and 75–100 µl of 1 M Tris-HCl (pH 9.0) to neutralize the pH.

For ADP analysis, 75 µl of sample was added with 125 µl of assay reagent consisting of (in mM unless otherwise stated) 38 Tris-HCl buffer (pH 7.4), 6.66 MgCl_2_, 0.12 M KCl, 1 phosphoenolpyruvate, 0.32 NADH, 57 U lactate dehydrogenase (LDH), and 43 U pyruvate kinase. For ATP analysis, 10 µl of sample was added to 190 µl of assay reagent consisting of (in mM unless otherwise stated) 38 Tris-HCl buffer, 6.66 MgCl_2_, 0.12 M KCl, 0.33 NADP+, 50 glucose, 1.26 U glucose-6-phosphate dehydrogenase, and 5 mU hexokinase. Both assay reactions were run to completion and as the amount of NADH was stoichiometric, ADP and ATP contents were determined per mg of protein as determined by the Biuret method with bovine serum albumin as standard.

### ATP production rate

A separate cohort of animals (n = 6 mice/group) were used to assess the net capacity of liver mitochondria to generate ATP on re-oxygenation and warming. Liver samples were stored in ice-cold UW solution and removed at 1.5, 5, 8, 12, 16 and 24 hours after storage. The liver sample was processed and placed in the Oxygraph as described above. Once a stable baseline oxygen flux was achieved, glutamate (10), malate (5), pyruvate (10) and succinate (10) were added. Then 10 µL of media was removed from each chamber and ADP (1.25) was added. The sampled aliquot was immediately mixed with 20 µL of ice-cold perchloric acid (3 M). Then 20 µL of 2 M KHCO_3_ was added before 190 µL of Tris-Base (1 M, pH 9.0) was added to neutralize the pH (7.5–8.0) and frozen in liquid nitrogen for future ATP analysis. Six more 10 µL samples were taken at 10 second intervals and treated similarly.

The aliquots were later thawed and centrifuged at 20,000 g for 1 minute (4°C). The supernatant ATP content was analyzed using a luciferase-based system (ATPlite, PerkinElmer, Massachusetts). The ATP synthesis rate was determined from the change in ATP content from sequential samples. This rate was divided by OXPHOS-CI,CII flux to provide a measure of ATP synthesis efficiency relative to amount of oxygen reduced (pmol ATP/pmol O_2_).

### Complex I & Complex II activities

Baseline hepatic CI and CII enzyme activities were determined from frozen samples using the NADH-oxidation and dichlorophenolindophenol-oxidation method, respectively [13]. Liver samples (20–50 mg) were homogenised in 1∶10 (wt/vol) ice-cold buffer (in mM: 210 mannitol, 70 sucrose, 5 HEPES, and 1 EGTA, pH 7.2). Homogenates were centrifuged for 10 minutes at 600 g (4°C) and the supernatant used for analysis. Assays were conducted using a 96-well format in a SpectraMax 340 plate reader (Molecular Devices, Sunnyvale) and results normalized to protein content determined by the Biuret method.

### Citrate synthase and lactate dehydrogenase activities

Citrate synthase activity was measured as a surrogate for mitochondrial mass [14]. Liver samples (10–50 mg) were weighed and homogenised in 1∶10 (wt/vol) ice-cold buffer (in mM, 50 KCl, 2 MgCl_2_, 1 EDTA, 25 Tris-HCl (pH 7.8) and 0.5% Triton X-100). Homogenates were centrifuged at 200,000 g for 10 minutes (4°C) and the supernatant removed for citrate synthase and LDH analysis. Citrate synthase activities were determined following Srere and modified to microtitre-plate [15]. LDH was measured to provide a measure of the anaerobic ATP synthesis capacity. Five µl of sample was added to 200 µl of assay reagent (in mM: 1 EDTA, 2 MgCl_2_, 1 DTT, 0.15 NADH and 0.15 pyruvate). The activity was determined from NADH-oxidation at 340 nm as pyruvate substrate was reduced to lactate [16].

### Liver tissue buffering capacity

Tissue buffering capacity of whole tissue and homogenate was compared between *ob/ob* and lean livers. A separate cohort of animals (n = 7 mice/group) was used to assess the pH change within the liver sample during cold ischemia. Liver samples were collected and stored in ice-cold UW solution. Two 3.5 mm pH probes (IQ240, Hach Company) were placed continuously in the UW solution and within the liver parenchyma, respectively, to measure the dynamic pH change over 24 hours of cold ischemia. Non-bicarbonate tissue pH buffering was measured in *ob/ob* and lean liver homogenate from the initial cohort of animals (n = 5 mice/group) to further compare tissue buffering capacity to acidosis. Tissue buffer capacity was determined between pH of 6.5–7.5 (20°C) as previously described [17]. Liver sections were weighed (95–108 mg) and homogenised in 20∶1 volume of 0.9% NaCl and transferred into a magnetically stirred 2-mL glass vial with a water jacket held at 20°C. The homogenate pH was adjusted to 6.5 with 0.2 M HCl and then titrated using a 10 µl Hamilton syringe up to pH 7.5 by sequential addition of 0.2 µM NaOH. The buffering capacity (β) was determined as β = µmol NaOH.Unit pH (6–7)^−1^.g wet wgt^−1^.

### Statistical and data analysis

All data are expressed as mean ± SEM. Statistical analyses were performed using GraphPad Prism version 5.0 (GraphPad Software, San Diego) for unpaired t-tests and two-way ANOVA; and SAS version 9.0 (SAS institute, Cary) for repeated measures analysis using restricted maximum likelihood with post-hoc Tukey-Kramer analysis. *P*<0.05 was considered statistically significant.

## Results

### *Ob/ob* mice are obese, glucose intolerant and insulin resistant

**Figure 1:**
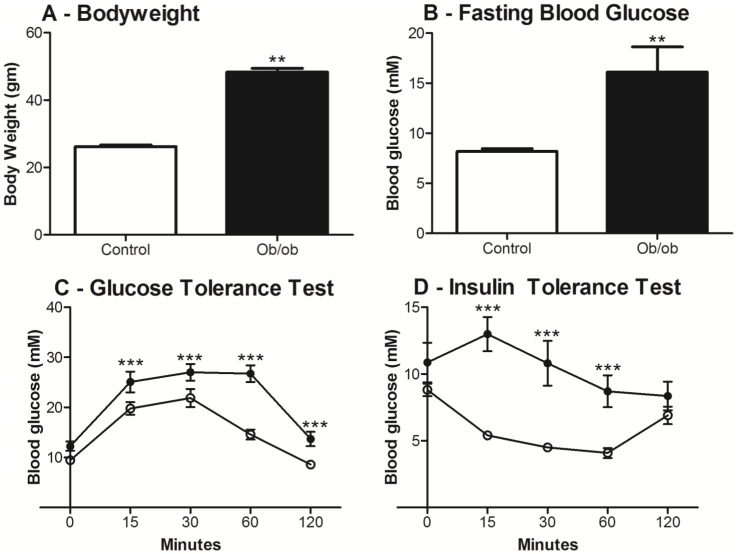
Body weight, fasting blood glucose, glucose tolerance test (GTT) and insulin tolerance test (ITT) of ten-week old *ob/ob* and lean mice. *Ob/ob* mice were significantly heavier (A) with significantly higher fasting blood glucose (B) than lean mice. GTT (C) and ITT (D) were performed after 12–13 hours of fasting. Compared to lean mice, *ob/ob* mice displayed glucose intolerance and insulin resistance. Data are shown as mean ± SEM (n = 9 mice/group; *ob/ob*, closed bar/closed circle; lean mice, open bar/open circle). Analyses were performed using Students t-tests for body weight and fasting blood glucose; and two-way ANOVA for GTT and ITT. **, *P*<0.01; ***, *P*<0.001.

Ten-week old *ob/ob* mice had increased body weight (48.3±1.1 vs. 25.3±0.3 g, *P*<0.01) and blood glucose level (16.1±2.5 vs. 8.2±0.3 mmol/L, *P*<0.01) relative to age-matched lean mice. *Ob/ob* mice also showed glucose intolerance (*P*<0.001) and insulin resistance (*P*<0.001) relative to aged-matched lean mice (Figure 1).

### *Ob/ob* mice livers had steatosis

**Figure 2:**
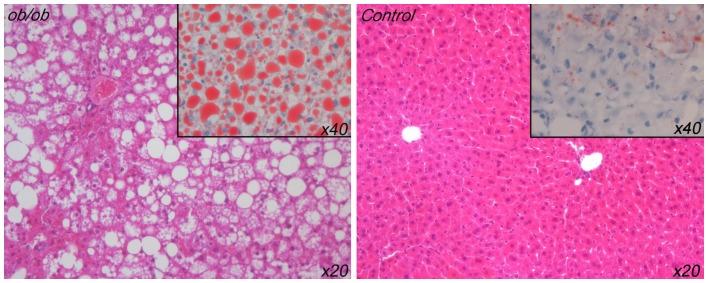
Liver tissue sections (4 µm) were stained for haemotoxylin and eosin (H&E, x20 magnification). *Insets*/sections (10 µm) were stained with Oil Red O (ORO, x40 magnification). Representative slides are displayed and *ob/ob* livers (left, n = 9) showed severe macrovesicular steatosis on both H&E and ORO. Lean livers (right, n = 9) showed no steatosis on H&E but ORO showed some patchy mild microvesicular steatosis.

The H&E staining showed that the lean mice livers had normal underlying tissue architecture whereas the *ob/ob* mice livers showed marked macrovesicular steatosis changes (Figure 2). The Oil Red O staining showed that the lean mice livers were predominantly normal, although they did display some isolated patchy red staining (up to 20–25%) that looked like a microvesicular steatosis pattern (Figure 2). This observation was however consistent with other reports of Oil Red O staining areas being observed in normal mice livers [18]. Conversely the *ob/ob* mice livers on Oil Red O staining showed widespread and consistently severe (>90%) macrovesicular steatosis, together with an associated minor component (<10%) of underlying microvesicular steatosis. These expected *ob/ob* findings confirmed the utility of the model and were also consistent with reports of significant hepatic steatosis in this same model by other researchers [19].

### Altered mitochondrial respiratory function in ob/ob liver

#### Impaired Complex I respiration

**Figure 3:**
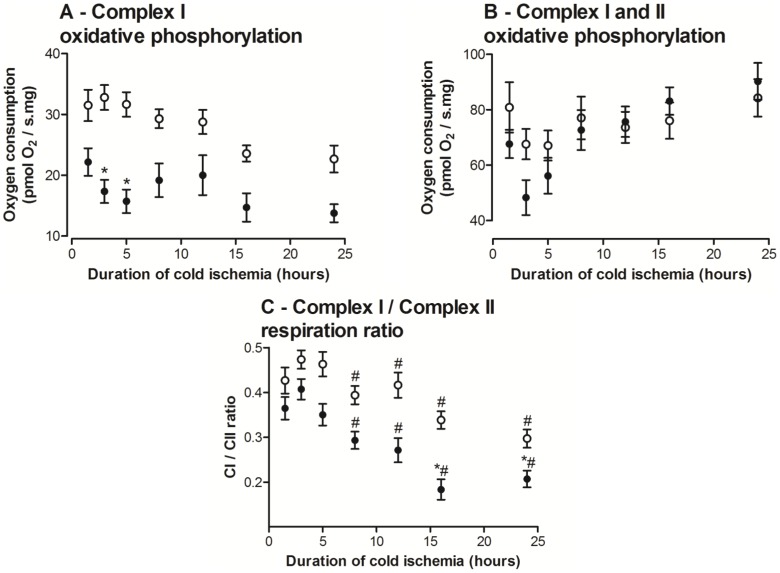
Mitochondrial function analysis of *ob/ob* and lean livers following different durations of cold ischaemia (hours). A: *Ob/ob* livers demonstrated lower Complex I (CI) oxidative phosphorylation throughout cold ischaemia and were significantly lower from lean livers at earlier time points. B: CI+Complex II (CII) oxidative phosphorylation did not differ between groups. C: After 8 hours of cold ischaemia, *ob/ob* and lean livers showed significantly lower CI respiration to CII respiration ratio compared to 3 hours of cold ischaemia. *Ob/ob* livers also showed significantly lower CI/CII ratios compared to lean livers after 16 hours of cold ischaemia. Data are shown as mean ± SEM (n = 9 mice/group; *ob/ob*, closed circle; lean livers, open circle). Restricted maximum likelihood with post-hoc Tukey-Kramer analysis was performed. *, *P*<0.05 (vs. lean); #, *P*<0.05 (vs. 3 hours).

OXPHOS-CI was lower in *ob/ob* livers at 1.5 hours compared to lean livers and remained lower at all time-points with the maximal significant difference at 3 and 5 hours (Figure 3A), which was only 53% and 50% that of the lean livers, respectively. The addition of exogenous Cytochrome *c* showed no significant flux increase indicating a functionally intact outer mitochondrial membrane in both groups. These results suggest there was an ETS impairment that progressively develops with cold storage duration, and it was significantly worse in steatotic livers.

#### Increased activation of Complex II respiration

OXPHOS-CI,CII flux was similar in both groups at 1.5 hours and remained stable over the duration of cold storage with no detectable difference between the groups (Figure 3B). This measures OXPHOS from both CI and CII, which more closely represents *in vivo* respiration where the majority of the ETS is functional. In the presence of rotenone (CI inhibitor), CII respirational flux was unchanged in both groups over time (Data not shown).

The OXPHOS-CI/OXPHOS-CI,CII ratio (Figure 3C) enables assessment of the simultaneous effects of functional changes in both CI and CII. OXPHOS-CI/OXPHOS-CI,CII ratio at 1.5 hours was lower in *ob/ob* livers compared to lean livers. While this ratio decreased over the duration of cold storage for both groups, it was consistently lower in *ob/ob* livers and was statistically significant by 16 hours of cold storage. The *ob/ob* livers indicate an earlier dependence on CII-based respiration supporting a likely underlying CI lability in *ob/ob* livers, which was accentuated by prolonged cold storage.

#### Decreased mitochondria efficiency

**Figure 4:**
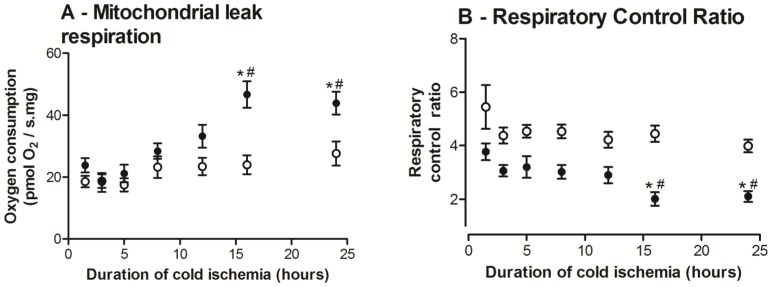
Mitochondrial leak respiration and respiratory control ratios (RCR) of *ob/ob* and lean livers following different duration of cold ischaemia (hours). A: *Ob/ob* livers showed increased mitochondrial leak rates with progressive cold ischaemia and increased significantly after 16 hours of cold ischaemia, while lean liver mitochondrial leak rates remained stable. B: *Ob/ob* livers consistently showed lower RCRs compared to lean livers and differed significantly after 16 hours of cold ischaemia. *Ob/ob* livers also demonstrated decreased RCR after 16 hours of cold ischaemia relative to 1.5 hours while lean liver RCR remained stable. Data are shown as mean ± SEM (n = 9 mice/group; *ob/ob*, closed circle; lean livers, open circle). Restricted maximum likelihood with post-hoc Tukey-Kramer analysis was performed. *, *P*<0.05 (vs. lean); #, *P*<0.01 (vs. 1.5 hours).

CI+CII leak (*L*_atra_) rate was assessed by addition of atractyloside (adenine nucleotide translocase inhibitor), which induces a respiration state dependent on proton leakage and specifically reflects mitochondrial membrane damage. *L*_atra_ at 1.5 hours was similar in both groups. However, *ob/ob* liver *L*_atra_ rates started to diverge after 8 hours of cold storage with two-fold higher leak rates after 16 hours of cold storage relative to baseline (Figure 4A), while lean liver *L*_atra_ rates were not significantly changed over time (*P* = 0.45). These data indicate that *ob/ob* liver mitochondria acquired more substantial and rapid mitochondrial membrane damage and proton leakage than lean livers.

*Ob/ob* livers had lower overall RCR means compared to lean livers at all time-points (Figure 4B). This became statistically significant after 16 hours of cold storage, where the *ob/ob* liver RCRs were 2.2 times lower than the corresponding lean livers. After 12 hours of cold storage, *ob/ob* livers also showed decreased RCR values relative to their baseline and this was statistically significant after 16 hours of cold storage. Lean livers demonstrated no significant change in RCR from baseline throughout the duration of cold storage. These data indicate that the *ob/ob* livers deteriorate slowly in the first few hours of cold storage, but this change increased considerably after 8–12 hours so that by 16 hours, they were significantly worse than lean livers.

#### No difference in maximal electron transport system capacity or Complex IV activity

ETS capacity and complex IV flux, which measures phosphorylation system control and Complex IV function, respectively, was similar between both groups throughout the period of cold storage (Data not shown). Additionally, these measures showed no significant change in either group at all time-points.

### No difference in baseline ATP content but decreased net ATP production rate in *ob/ob* livers following cold ischemia

**Figure 5:**
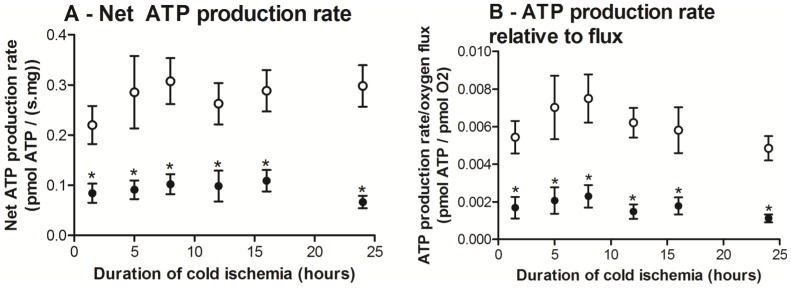
Net adenosine triphosphate (ATP) production rate and ATP production rate relative to flux of *ob/ob* and lean livers following different duration of cold ischemia (hours). *Ob/ob* livers showed significantly lower net ATP production (A) and ATP production rate relative to flux (B) at all time points. Data are shown as mean ± SEM (n = 6 mice/group; *ob/ob* livers, closed circle; lean livers, open circle). Analyses were performed using two-way ANOVA. *, *P*<0.05 (vs. lean).

Basal hepatic ADP and ATP contents (Table 1) were similar between the groups, which indicated that despite lower CI respiration, *ob/ob* livers maintained similar ATP levels *in vivo*. However, net ATP production rate of *ob/ob* livers was lower at all time-points (Figure 5A), indicating decreased ATP regeneration capacity on reperfusion after a cold ischemic insult. ATP production efficiency (pmol ATP/pmol O_2_) were significantly lower in *ob/ob* livers (Figure 5B). Therefore, for every oxygen molecule consumed, *ob/ob* livers have a significantly lower net ATP production capacity.

**Table 1:** Baseline measurement of ADP and ATP contents; Complex I, Complex II, Citrate Synthase and lactate dehydrogenase levels in *ob/ob* and lean livers.

	*Ob/ob* livers	Lean livers
**ADP (nmol/mg protein)**	**3.96±0.29**	**3.93±0.75**
**ATP (nmol/mg protein)**	**8.81±1.60**	**8.70±1.20**
**Complex I (nmol/min per mg protein)**	**145.3±34.8** *	**267.5±37.2**
**Complex II (nmol/min per mg protein)**	**222.4±35.2**	**281.3±40.9**
**Citrate Synthase (milliunit/min per mg protein)**	**473.1±69.1**	**493.6±25.1**
**Lactate dehydrogenase (Unit/mg protein)**	**2.38±0.31** **	**5.72±0.38**
**Protein content (µg/mg tissue)**	**53.3±12.6**	**43.3±6.8**

ADP, Adenosine diphosphate; ATP, Adenosine triphosphate; Data are expressed as mean ± SEM (n = 9 mice/group).

*P<0.05 vs. lean livers;

**P<0.01 vs. lean livers.

### Assessment of mitochondrial content and ETS complex activities

There was no difference in mitochondrial protein content between the two groups (Table 1). Baseline citrate synthase activity was also similar between the groups (Table 1), indicating similar mitochondrial content in both groups. The *Ob/ob* livers had significantly lower baseline CI activities but similar basal CII activities as lean livers (Table 1).

### *Ob/ob* livers have lower anaerobic capacities and tissue pH buffering capacities

**Figure 6:**
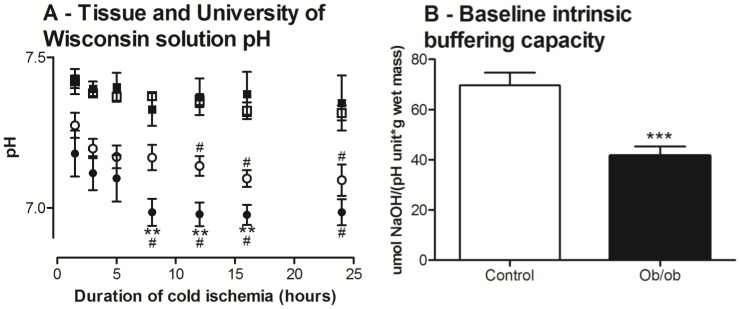
University of Wisconsin (UW) solution and tissue pH of *ob/ob* and lean livers during cold ischemia; and baseline intrinsic tissue buffering capacity of *ob/ob* and lean livers. A: UW solution pH (*ob/ob*, closed square; lean, open square) remained stable during the whole duration of cold ischemia and did not differ between groups. However, *ob/ob* livers demonstrated significantly lower tissue pH following 8–16 hours of cold ischemia compared to lean livers. *Ob/ob* and lean livers demonstrated decreased tissue pH after 8 and 12 hours of cold ischemia, respectively, relative to 1.5 hours. B: *Ob/ob* livers also demonstrated significantly lower baseline tissue intrinsic pH buffering capacity. Data are shown as mean ± SEM (n = 9 mice/group; *ob/ob*, closed circle/bar; lean, open circle/bar). Analyses were performed using two-way ANOVA for tissue pH and un-paired t-test for intrinsic tissue buffering capacity. **, *P*<0.01 (vs. lean); ***, *P*<0.001 (vs. lean); #, *P*<0.01 (vs. 1.5 hours).

*Ob/ob* livers had lower baseline LDH activities (Table 1), indicating an inherent decreased capacity for anaerobic ATP generation and reduction-oxidation maintenance. Measurements of pH of the UW solution surrounding the stored livers were similar between the groups and remained stable throughout 24 hours of cold ischemia (Figure 6). However, pH within the stored liver tissue itself declined 30% faster in the *ob/ob* livers than in lean livers (Figure 6; *P*<0.05) and was more acidotic (6.98 vs. 7.17; *P*<0.05) after 8 hours of storage. This finding was confirmed when we found the intrinsic liver tissue pH buffering capacity of *ob/ob* livers at baseline was 40% lower than lean livers (Figure 6; *P*<0.01). These data indicated that *ob/ob* livers were not capable of buffering acidosis as effectively as lean livers.

## Discussion

In this study, we used *ob/ob* mice with established hepatic macrovesicular steatosis. *Ob/ob* livers demonstrated decreased baseline CI activity but similar baseline ATP contents compared to lean livers. This study outlines a detailed assessment of mitochondrial function in fatty livers, and was undertaken to characterize the interactions between cold storage and duration of preservation. *Ob/ob* liver mitochondria had accelerated time-dependent cold ischemic damage compared to normal liver. This was illustrated by a 2-fold increased proton leak and a two-fold decreased mitochondrial efficiency, resulting in impaired ATP production upon re-oxygenation after cold storage. *Ob/ob* liver also had lower anaerobic capacities, indicating impaired substrate level phosphorylation capacities to synthesize ATP. *Ob/ob* tissues were found to have attenuated intrinsic tissue pH buffering capacities making it prone to increased damage from acidosis. These data demonstrate that *ob/ob* livers developed significant underlying impairments in mitochondrial aerobic and anaerobic ATP production capacities, which were further exacerbated by cold storage ischaemia. This study was the first study to investigate the impact of hepatic steatosis on mitochondrial function between the time-points of 9 and 18 hours of cold ischemia.

We focused on MF as it is crucial for normal cellular function as it generates 95% of cellular ATP in aerobic states and interruption of mitochondrial processes can disrupt cellular bioenergetics leading to cell death [6]. During ischemia, cellular ATP levels significantly decreases [4], [20] and following reperfusion, adequate MF providing sufficient ATP plays a key role in post-ischemic cellular recovery [21]. Hepatic steatosis is currently the most common hepatic abnormality encountered during liver surgery [1]. There is however emerging evidence that steatotic livers have impaired MF and this is associated with the increased susceptibility to ischemia-reperfusion injury [7], [22]. In this study we have investigated in more detail how normal hepatic MF was altered by steatosis and cold storage.

In this study, we confirmed that the *ob/ob* mice model demonstrated systemic characteristics of the metabolic syndrome with marked obesity, hyperglycaemia, glucose intolerance and insulin resistance (Figure 1). It is acknowledged however that like most animal research disease models, it has limitations. In particular the *ob/ob* model is based on a genetic leptin deficiency that drives hyperphagia, but that underlying etiology is not considered a common cause in human hepatic steatosis pathophysiology [23]. The *ob/ob* liver responses to cold preservation may therefore not entirely reflect all the responses of the human steatotic liver state. The *ob/ob* mice model is nonetheless considered a useful model of hepatic steatosis and is widely used in steatotic research studies [19], [22].

The *ob/ob* mice in our study showed consistent high grade macrovesicular steatosis on histology by both H&E and Oil Red O staining. In contrast, the lean mice livers demonstrated normal tissue architecture on H&E staining. They also showed largely normal appearances on Oil Red O staining, although they did have some patchy background microvesicular-like staining. This might have represented some degree of mild microvesicular steatosis; however some caution is needed in interpreting this observation. Oil Red O staining has the potential to “over-estimate” hepatic steatosis, as supported by the report of prominent sinusoidal Oil Red O staining in ‘normal’ human livers [24]. Additionally, normal lean mice livers have been reported to show multiple small, but clearly Oil Red O positive lipid droplets [18]. This is similar to the findings in our study. Furthermore, we note that microvesicular steatosis is associated with normal outcomes in liver surgery and is considered to be of little significance to post-operative graft outcome [25], [26]. These observations are consistent with the normal physiological status of our own lean control mice. Taken together, our findings and the literature suggest that some degree of underlying Oil Red O microvesicular staining may be part of the spectrum of normal mice livers.

### Complex I

CI is fundamentally important to the mitochondrial ETS and impaired CI respiration has a substantial impact on ATP synthesis [27]. We observed lower baseline CI activity in *ob/ob* livers by 46% and investigated how this altered during cold storage. The data showed progressive CI impairment as well as an increasing dependence of *ob/ob* livers on CII respiration during cold storage. As steatotic livers produce more reactive oxygen species *in vivo* [28], they may potentially down-regulate CI activity to limit reactive oxygen species generation and decrease mitochondrial damage, or more CI molecules are damaged. Rat hearts subjected to ischemia showed decreased CI activities, a response thought to result from reactive oxygen species damage to cardiolipin (mitochondrial inner membrane phospholipid) [29]. Similar effects may be accentuated in steatotic livers. In addition mitochondrial membrane alterations that permit a decreased mitochondrial membrane potential could impede the electrogenic glutamate and pyruvate uptake, which all support CI flux [11]. While these explanations may account for the lower baseline CI activities within steatotic livers, when steatotic livers were subjected to a severe insult (prolonged cold storage), further decreases in CI function were observed, indicating further insults from any of the mechanisms suggested above.

### Complex II

CII is the only mitochondrial membrane-bound enzyme involved in the citric acid cycle [30]. Our baseline CII results are consistent with a previous study that reported similar baseline CII enzyme activity in *ob/ob* and lean livers [22]. We also observed similar CII function in steatotic and lean livers throughout prolonged cold ischemia, consistent with reports that CII abnormalities are infrequently reported [13]. Additionally, intact CII function has been shown previously in cold storage of steatotic livers [7]. CII-mediated flux generates less ATP relative to CI-mediated flux, thus succinate-fuelled respiration provides a less efficient ATP generation mechanism. This may explain why CII activity cannot compensate for the impaired CI and further explain some of the decreased ATP production rate in steatosis, which adds to the slower recovery of steatotic livers post-transplantation.

### ATP synthesis capacity

Despite underlying MF impairment, *ob/ob* livers were able to maintain an adequate cellular ATP level *in vivo*. While some studies showed decreased baseline ATP levels within steatotic livers [19], [22], others reported similar ATP levels compared with lean livers [7]. However, under transplant preservation settings, ATP levels are rapidly depleted during prolonged cold ischemia of 18 hours [7] but there have been no studies before this time-point. Therefore intact MF on reperfusion becomes paramount to cellular survival by providing rapid ATP re-synthesis to cope with the impact of reperfusion injury. On re-oxygenation following cold ischemia, *ob/ob* livers had significantly lower net ATP production rates in the presence of excess substrates and supra-physiological oxygen, a result that now explains other studies that reported lower post-reperfusion steatotic liver ATP content compared to lean livers [19], [22]. We extended this to show that ATP production was depressed in *ob/ob* livers regardless of cold storage times.

The mechanism for the impaired ATP production rate is likely to be multi-modal. As well as attenuated CI activity, we showed there were alterations to the inner membrane integrity as shown by the increased *L*_Atra_ respiration, consistent with some who have reported that isolated *ob/ob* liver mitochondria have greater proton leak rates relative to lean liver mitochondria [31]. Increased membrane leakiness may be due to altered mitochondrial membrane composition. Increased mitochondrial cholesterol loading was shown to lead to decreased mitochondrial membrane fluidity with impaired adenonucleotide translocase function [32], suggesting a difference in the mitochondrial membrane composition in steatosis may play a role in the response to cold ischemia. Our finding of depressed *ob/ob* liver RCR values strongly support a leakier inner mitochondrial membrane, which leads to a decreased ability to rapidly recover ATP synthesis capability in the critical reperfusion phase. Importantly our data were generated using permeabilised tissues, a method shown to better reflect *in vivo* MF [10]. Furthermore, the only study into steatotic liver mitochondrial function during cold ischemia was performed at a non-physiological temperature of 30°C and in isolated mitochondria [7]. Mitochondrial respiration is temperature-dependant, in particular LEAK state respiration, which decreases relative to phosphorylating respiration flux (and therefore raises the RCR) [33]. The process of mitochondrial isolation disrupts mitochondrial structure and introduces bias by preferential selection of healthy mitochondria [34]. Our study adjusts for these confounding factors by analyzing mitochondrial function at 37°C and in permeabilised tissues.

Despite the described CI impairment and increased proton leak, other ETS aspects appear intact in *ob/ob* liver mitochondria. Our findings of similar ETS capacities between steatotic and lean livers are consistent with another study [7], and suggests that *ob/ob* liver mitochondria have at least a similar turnover of oxygen. However, our evidence of lower CI activity would suggest that the flux contributions from CI and CII at the Q-junction [35] differ. Additionally, citrate synthase activity was similar across groups, indicating that MF differences results from functional mitochondrial impairment and not decreased mitochondrial mass. We note that this study did not measure specific mitochondrial substrate oxidation function and so future studies could consider measurement of these to give a more complete perspective of the mitochondrial response to cold ischemia.

### Anaerobic metabolism and tissue buffering capacity

During anoxia, anaerobic glycolysis generates ATP through reduction of pyruvate to lactate by LDH, and this is important during prolonged cold storage [36]. However, sustained and inefficient anaerobic glycolysis promotes acidosis, and acidosis inactivates glycolytic enzymes through the Pasteur effect to further suppress ATP synthesis [37]. Protons released from ATP hydrolysis together with attenuated mitochondrial and anaerobic capacities in steatotic livers (less LDH) will further promote acidosis as ATP production consumes protons. Under ischemic conditions, the usual major bicarbonate pH buffering system cannot operate due to lack of clearance by local venous perfusion and pulmonary carbon dioxide removal. Ischemic hepatocytes must rely on other intracellular di-peptides and protein buffering agents such as those containing L-histidine [37]. Our study was the first to demonstrate that *ob/ob* livers had impaired acid buffering capacities compared to lean livers and generated a greater amount of proton accumulation during cold ischemia. This may have implications for further research into cold storage for steatotic livers.

Tissue acidosis is a key inhibitor of respiration and is most extensively studied in skeletal muscle where mitochondria show depressed OXPHOS, and elevated LEAK fluxes following exposure to lactate-induced acidosis at physiologically relevant levels (pH 6.38, 8 mM) [38]. Mitochondrial membrane potentials may also decrease with acidosis [38]. Thus proton accumulation may account for the elevated leak respiration and depressed RCRs observed in steatotic *ob*/*ob* livers, which is consistent with damaged inner mitochondrial membranes. The transplantation situation presents an additional challenge for the poorly pH buffered steatotic livers, as unlike skeletal muscle, livers must accommodate their own internally derived acidosis, as well the systemic lactate accumulation from the anhepatic period [39]. On reperfusion lactate is mostly cleared aerobically by the liver and insufficient hepatic lactate clearance is a primary indicator of liver graft non-function [39] .

Currently, there are no formal guidelines on steatotic donor liver allocation but prolonged cold ischemia is avoided for steatotic livers [1]. A recent study of 5051 patients post-transplantation indicated that >30% macrovesicular steatosis, cold ischemia of >5 (≤11) hours and >11 hours were all independent risk factors associated with graft loss [40]. Our results suggest that steatotic rodent liver MF started to deteriorate between 5–8 hours of cold ischemia. This supports a policy of shorter cold ischemic times if using a steatotic liver donor [40]. Although the inherent CI dysfunction would still be present, shortening the cold ischemic time to <5 hours may prevent the development of membrane leak, an avoidable acquired MF damage during prolonged cold ischemia. Additionally, the shortened cold ischemic time may also forestall steatotic liver tissue acidosis and prevent further acidosis-related impairment of mitochondrial respiration.

In this study we have provided a new detailed analysis of the interaction between steatosis and MF. With the increasing prevalence of marginal donors, improving our understanding of the underlying mechanism of steatotic liver susceptibility to cold ischemic damage is critical. Our results highlighted the role of MF and ATP production in the susceptibility of steatotic livers to cold ischemia. Steatotic rodent livers develop inherent MF impairment and further acquire time-dependant mitochondrial damage following prolonged cold ischemia that was multi-factorial. A key time-point has emerged that damage accelerates after 5–8 hours of cold storage with an upper threshold of 12 hours. We can now offer a mechanistic rationale for the use of shortened cold ischemic times and prevention of pathological mitochondrial leak. Further studies into the nature of acquired ETS complex damage from steatosis and cold storage are now planned.
